# Preference for Object Relative Clauses in Chinese Sentence Comprehension: Evidence From Online Self-Paced Reading Time

**DOI:** 10.3389/fpsyg.2019.02210

**Published:** 2019-10-01

**Authors:** Kunyu Xu, Jeng-Ren Duann, Daisy L. Hung, Denise H. Wu

**Affiliations:** ^1^Institute of Cognitive Neuroscience, National Central University, Taoyuan, Taiwan; ^2^Institute for Neural Computation, University of California, San Diego, La Jolla, CA, United States; ^3^College of Humanities and Social Sciences, Taipei Medical University, Taipei, Taiwan

**Keywords:** Chinese relative clause, self-paced reading, ORC preference, dependency locality theory, integration cost

## Abstract

Most prior studies have reported that subject-extracted relative clauses (SRCs) are easier to process than object-extracted relative clauses (ORCs). However, whether such an SRC preference is universal across different languages remains an open question. Several reports from Chinese have provided conflicting results; thus, in the present study, we conducted two self-paced reading experiments to examine the comprehension of Chinese relative clauses. The results demonstrated a clear ORC preference that Chinese ORCs were easier to comprehend than Chinese SRCs. These findings were most compatible with the prediction of the integration cost account, which claims that the processing difference between SRCs and ORCs arises at the point of dependency formation. The ORC preference in Chinese poses a challenge to the universality of the SRC preference assumed by the structural distance hypothesis and highlights the values of cross-linguistic research.

## Introduction

Empirical cross-linguistic research on different aspects of sentence processing has provided evidence for the universality and specificity of linguistic processing mechanisms. Researchers investigating cross-linguistic syntactic processing have studied relative clause (RC) structures. Because most languages have RC sentences, such materials provide an opportunity to investigate the universality of the processing mechanisms across languages (Keenan and Comrie, 1977).

There are different types of RCs in languages. Based on the syntactic role of the head noun being modified by RCs, RCs are mainly classified into subject-extracted relative clauses (SRCs) and object-extracted relative clauses (ORCs). SRCs and ORCs differ minimally in the surface form but substantially in the syntactic structure. In the previous literature, research on a wide range of languages has frequently found that there is a preference for SRCs over ORCs when RCs contain full noun phrases (NPs), such as English (e.g., King and Just, 1991; King and Kutas, 1995; Stromswold et al., 1996; Gibson, 1998; Traxler et al., 2002; Grodner and Gibson, 2005), Dutch (e.g., Frazier, 1987; Mak et al., 2002), French (e.g., Holmes and O’Regan, 1981), and German (e.g., Mecklinger et al., 1995). In English, one exception to the general SRC advantage is when the RC contains a personal pronoun (e.g., ‘the people that you like’ vs. ‘the people that like you’). The less common preference for ORCs over SRCs in this case might be due to the higher frequency of the former than the latter structure (Reali and Christiansen, 2007). Findings against the SRC preference in some languages such as Chinese (e.g., Hsiao and Gibson, 2003; Chen et al., 2008; Gibson and Wu, 2013; Sung et al., 2016) and Basque (Carreiras et al., 2010) have also been reported. Even within the same language, such as Chinese, the preference for SRCs (e.g., Lin and Bever, 2006) or ORCs (as cited above) is still paradoxical as shown in conflicting results. Considering that Chinese is a language with the unique combination of subject-verb-object word order and a head-final property (e.g., Dryer, 1992; Lin, 2008; Wu et al., 2012), revealing its RC processing preference is important to examine the universality of the SRC preference that is dominant in the literature.

### Theoretical Accounts for Relative Clause Processing

In parallel with the perplexing results observed in behavioral experiments, various theoretical accounts also make different predictions about Chinese RC processing preference.

#### Structural Distance Account

**FIGURE 1:**
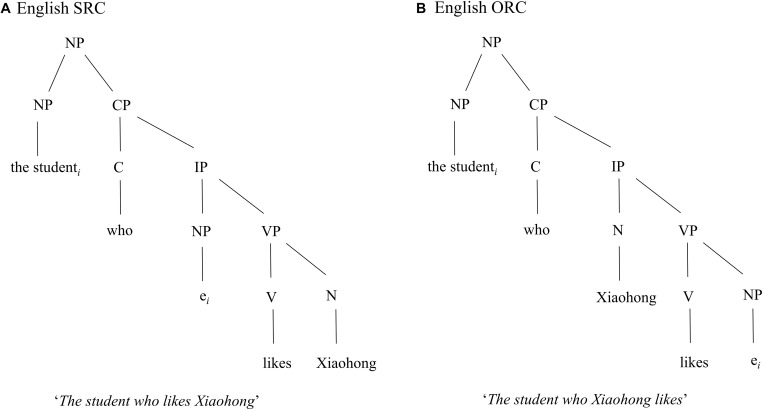
The syntactic structures of an example of English SRC and ORC sentences. As shown in the figure, there are more syntactic nodes (NP, NP, CP, IP, VP, NP) intervening between the gap *e* and the head noun (i.e., the student) in the ORC **(B)** than the nodes (NP, NP, CP, IP, NP) in the SRC **(A)**, meaning that the English ORC is predicted to be more difficult to process than the English SRC by the structural distance account.

According to the structural distance account put forward by O’Grady (1997), the longer the structural distance between the filler and the gap is, the more complex the sentence is. During the parsing process, the structural distance is defined as the number of syntactic nodes/projections that intervene between the filler and the gap in the syntactic tree. As shown in Figure 1, in an SRC, the subject-gap position is within the inflection phrase (IP), whereas in an ORC the object-gap is embedded in the verb phrase (VP), which is deeper than the IP in the syntactic tree. Thus, there are more syntactic nodes (NP, NP, CP, IP, VP, NP) intervening between the gap *e* and the head noun (i.e., the student) in the ORC than the nodes (NP, NP, CP, IP, NP) in the SRC.

**FIGURE 2:**
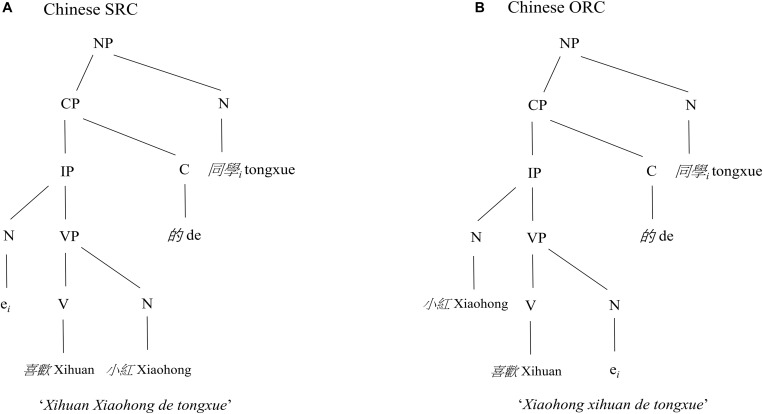
The syntactic structures of an example of Chinese SRC and ORC sentences. As shown in the figure, there are more syntactic nodes (N, NP, CP, IP, VP, N) between the head noun (i.e., *tongxue*) and the object gap *e* in the Chinese ORC **(B)**, thus a deeper structure, than the nodes (N, NP, CP, IP, N) between the same head noun and the subject gap *e* in the Chinese SRC **(A)**, hence a preference for Chinese SRCs over ORCs is hypothesized by the structural distance account.

The structural distance account predicts that the sentences with a larger number of syntactic nodes are more difficult to process than the sentences with a smaller number of syntactic nodes in a syntactic tree. As a result, an SRC preference is predicted for English. Moreover, this account assumes that the underlying syntactic structure is universal across languages. Thus, a preference for SRCs over ORCs is hypothesized for all languages. Following this account, in a Chinese ORC the distance (N, NP, CP, IP, VP, N) between the head noun (i.e., *tongxue*) and the object gap *e* is greater, hence structurally deeper, than the distance (N, NP, CP, IP, N) between the same head noun and the subject gap *e* in a Chinese SRC as illustrated in Figure 2.

#### Memory-Based Account

One prominent example among the memory-based accounts is the Dependency Locality Theory (DLT) proposed by Gibson (1998). According to the DLT, processing difficulty is mainly due to working memory constraints on sentence comprehension. Specifically, the DLT claims that a sentence is parsed based on two metrics: the storage cost and the integration cost. The storage cost is related to the process of maintaining temporarily incomplete dependencies during sentence processing. The integration cost, on the other hand, is related to the process of establishing connections between the incoming words and the current syntactic structure (Gibson, 1998). The processing difficulty is assumed to increase with a large number of new discourse references that intervene between the element that is currently being processed and the elements with which a syntactic dependency has to be built. For English, both the storage and the integration costs are greater in an ORC than in an SRC. Specifically, incomplete head-dependencies in an ORC are more distant than those in an SRC. Therefore, the storage demand to keep track of the syntactic heads that are needed to form a grammatical sentence is greater in the former than in the latter case. For syntactic integration, on the other hand, the object-subject-verb word order of English ORCs demands non-local integration (see also Gordon and Lowder, 2012), resulting in a greater integration cost in processing English ORCs than SRCs. That is, an SRC preference is hypothesized for English by both the storage and integration costs of the DLT.

Different from the prediction of an SRC preference for English, an ORC preference is predicted for Chinese according to the DLT. During the comprehension of the Chinese SRC, rènshi Zhāngsān de sījī (‘*The driver who knew Zhangsan*’), the three syntactic heads before the noun *sījī* (i.e., rènshi, Zhāngsān and de) need to be stored in working memory. On the other hand, only one predicted head is required during the comprehension of the Chinese ORC, Zhāngsān rènshi de sījī (‘*The driver who Zhangsan knew*’). Thus, a smaller number of temporarily incomplete dependences results in a lower storage cost in a Chinese ORC than a Chinese SRC. As for the integration cost, when the relativizer *de* and the head noun are encountered, a greater processing demand is required to complete the integration across a longer filler-gap distance in an SRC whose word order is non-canonical (object-verb-subject), compared with that in an ORC whose word order is canonical (subject-verb-object). Therefore, according to both the storage and integration costs of the DLT, SRCs should be more difficult to comprehend than ORCs in Chinese.

#### Experience/Frequency-Based Accounts

The experience-based account, proposed by Mitchell et al. (1995), suggests that the human sentence parser is experience-based, and the relative frequency of each type of relative clauses determines its relative processing difficulty. For English, some corpus statistics have found that SRCs with full NPs are more frequent than ORCs with full NPs; thus, the latter structure is more difficult to process than the former structure (Roland et al., 2007). MacDonald and Christiansen (2002) employed a Simple Recurrent Network (SRN) model to investigate the importance of learning or experience to RC comprehension. The results indicated that distributional constraints have effects on comprehension of SRCs and ORCs in English. That is, processing of SRCs (with full NPs) benefited from many simple transitive sentences that shared the overwhelmingly frequent word order (subject-verb-object), while processing of ORCs benefited almost exclusively from direct experience with ORCs themselves. Based on such results, Wells et al. (2009) further manipulated participants’ reading experience on RC construction and found that the reading pattern of RCs strikingly resembled the results from MacDonald and Christiansen’s (2002) computational model, showing reduced reading times more for ORCs than SRCs after equivalent exposure to the respective RC structures. Besides, Reali and Christiansen (2007) conducted a series of behavioral experiments to examine the role of experience in sentence processing by investigating the processing difference between pronominal SRCs and ORCs. They found that highly frequent pronominal ORCs, as revealed by a large-scale corpus analysis, were indeed easier to process than pronominal SRCs when the embedded pronoun was personal. These results together highlighted the contribution of experience to RC processing in mature readers and provided strong support for experience-based approaches (see Christiansen and Chater, 2016 for more details).

For Chinese, corpus studies have reported that Chinese SRCs are more frequent than Chinese ORCs, thus predicting an SRC advantage in Chinese (Pu, 2007; Vasishth et al., 2013). However, Hsiao and MacDonald (2013) adopted a similar SRN model as that employed in MacDonald and Christiansen (2002) and found that sentence difficulty might not be simply determined by sentence frequency. Specifically, they found that ORCs yielded fewer errors than SRCs did, especially at the head noun region. Although such results were not predicted by the frequency-based accounts, they were consistent with previous studies that reported faster reading times for ORCs than SRCs (e.g., Hsiao and Gibson, 2003; Gibson and Wu, 2013).

### Previous Findings of the Processing Preference for Chinese Relative Clauses

The first study to explore the Chinese RC preference was conducted by Hsiao and Gibson (2003). They found that ORCs were easier to comprehend than SRCs when both types of RCs were at the subject-modifying position, particularly being reflected in the self-paced reading time of the embedded clause region. These results are regarded as evidence for the storage cost account of the DLT. On the contrary, Lin and Bever (2006) claimed that there was no significant preference between the reading of Chinese SRCs and ORCs at the subject-modifying position. Rather, they reported an SRC preference only at the object-modifying position. However, as Gibson and Wu (2013) pointed out, the SRC preference observed for object-modifying RCs might be due to local syntactic ambiguity (see similar ERP findings in Bulut et al., 2018). To overcome this potential problem, Gibson and Wu (2013) designed a disambiguating preceding context to minimize the garden path effect in sentences. Consistent with their earlier results (Hsiao and Gibson, 2003), SRCs were read significantly more slowly than ORCs, which again was in line with the prediction of the DLT. Vasishth et al. (2013) later attempted to replicate the results from Hsiao and Gibson (2003) and Gibson and Wu (2013) by conducting three self-paced reading experiments. Similar to the conflicting results in the literature, two of their experiments showed the SRC preference, while the other one presented the ORC preference. Based on a meta-analysis of 15 previous RC studies in Chinese (including their own experiments), Vasishth et al. (2013) claimed that the SRC preference was more dominant than the ORC preference. Thus, their results were against the predictions of the DLT but supported the experience-based account. In addition to syntactic structure and memory demands, the factor of thematic order was also found to affect Chinese RC processing. Lin (2014) employed similar materials with preceding disambiguating contexts as in Gibson and Wu (2013) and found that the comprehension of Chinese RCs was sensitive to the thematic role orders both in the preceding discourse context and in the subsequent RC. Specifically, the PATIENT-AGENT-action order of a passive sentence in the preceding discourse context (e.g., lìngyíwèi zhùhù zé bèi zhèwèi fángdōng chǎoxǐngle, ‘*the other tenant was woken up by the landlord*’) did not facilitate either the action-PATIENT-AGENT order of an SRC (e.g., chǎoxǐng fángdōng de zhùhù, ‘*the tenant that woke up the landlord*’) or the AGENT-action-PATIENT order of an ORC (e.g., fángdōng chǎoxǐng de zhùhù, ‘*the tenant that the landlord woke up*’). However, when the preceding discourse context (e.g., zhèwèi fángdōng zé chǎoxǐngle lìngyíwèi zhùhù, ‘*the landlord then woke up the other tenant*’) presented a thematic order consistent with the subsequent ORC (e.g., fángdōng chǎoxǐng de zhùhù, ‘*the tenant that the landlord woke up*’), the Chinese ORC was read faster than the Chinese SRC.

In addition to findings from mature readers, the processing of RCs during language acquisition can also shed light on the processing asymmetry between SRCs and ORCs. The results from English- and German-speaking children were consistent with previous studies in showing an advantage for SRCs over ORCs (Diessel and Tomasello, 2005). However, the disadvantage of ORCs was found to be mitigated and even eliminated when the subject was pronominal and the direct object was inanimate (Kidd et al., 2007). For Chinese, similar to the controversial findings from adults, the results from children were also mixed. Chang (1984) used an act-out task to test preschool Mandarin-speaking children on their comprehension of different types of RCs, but the results pointed to neither an SRC or ORC advantage. Hsu et al. (2009) then used an elicited production task and found that Chinese SRCs were easier to comprehend than ORCs for children. However, Su (2006) failed to identify a clear SRC advantage with the same task. Afterward, Chan et al. (2011) adopted a picture-pointing task and reported an ORC preference during children’s acquisition of RC structures. Despite the recent behavioral and developmental research on Chinese RC processing, the processing preference between SRCs and ORCs remains undetermined.

### Neurophysiological Studies on Relative Clause Processing

The advances of techniques have allowed new approaches to examine the processing difficulty of SRCs and ORCs. Neurophysiological measurements provided the evidence in support of an SRC advantage in English by showing that the processing of both written and spoken ORC sentences elicited greater negative waveforms than SRC sentences at the ‘gap’ position of the sentence in single-word ERPs, while only the SRC but not ORC sentences elicited a slow frontal positivity at the multiword level, which was interpreted as an index of ease of processing or integration (King and Kutas, 1995; Muller et al., 1997). On the other hand, Weiss et al. (2005) examined large-scale oscillatory activity during the comprehension of English SRCs and ORCs by using EEG coherence analysis and also found a continuous higher coherence for ORCs than SRCs in the theta, beta and gamma frequency bands, which were suggested to be associated with memory processes, attentional effort and semantic-pragmatic integration, respectively. In contrast with the generally consistent findings of an SRC advantage in English, the relatively scarce evidence on Chinese RC processing seems to support an ORC advantage. The neurophysiological studies have identified greater ERP components elicited by SRCs than ORCs (Yang and Perfetti, 2006; Packard et al., 2010; Yang et al., 2010), though the time windows of the observed effects might vary in different studies (see Bulut et al., 2018, for a summary).

### The Current Study

Following the earlier research on the RC processing preference, an increasing number of empirical studies have examined the factors that influence the reading of Chinese RCs, including thematic order (e.g., Gibson and Wu, 2013; Lin, 2014), animacy (e.g., Wu et al., 2012; He and Chen, 2013), relative frequency occurrence (e.g., Vasishth et al., 2013), discourse context (e.g., Yang and Perfetti, 2006; Gibson and Wu, 2013), and working memory (e.g., Hsiao and Gibson, 2003). Furthermore, the compounding effects caused by different aspects of language (i.e., syntax, semantics, and pragmatics) might reflect multiple mechanisms that contribute to the RC processing preference. In the present study, to determine the contribution of the syntactic structure to the RC processing, we focused on the basic form of Chinese RC sentences (as shown below) by employing the self-paced reading task. The self-paced reading task has been widely used to observe online reading time and offline performance to probe questions in sentence comprehension (e.g., Just et al., 1982; King and Just, 1991). As introduced above, the structured-based and experience-based accounts predict an SRC advantage in Chinese, while the DLT proposes an ORC advantage in Chinese. It is expected that the processing time for the embedded clause region of SRCs should be significantly longer than that of ORCs according to the storage cost account of the DLT. On the other hand, longer processing time on the relativizer and/or the head noun of SRCs than that of ORCs is predicted by the integration cost account of the DLT. We originally conducted three self-paced reading experiments to examine these predictions with subject- and object-modifying RCs separately. Because the reading preference of subject-modifying RCs predicted by the integration cost accounts such as the DLT would be identical to that resulting from ambiguity resolution, however, the conclusion from subject-modifying RCs would be less interpretable than that from object-modifying RCs. Therefore, we reported the findings from two experiments on object-modifying RCs in the main text below, while describing the results from one experiment on subject-modifying RCs in the Appendix.

## Experiment 1

Experiment 1 was designed to investigate the processing preference between Chinese SRCs and ORCs at the object-modifying position.

### Method

#### Participants

Forty-six native Chinese-speaking students (26 females, aged from 20 to 28 years) from National Central University participated in this experiment. All participants reported being right-handed with a normal or corrected-to-normal vision. The study was carried out in line with the recommendations of the Social and Behavioral Research Ethical Principles and Regulations of National Taiwan University. All participants gave written informed consent in accordance with the Declaration of Helsinki. The protocol was approved by the Research Ethics Committee of National Taiwan University.

#### Stimuli

Sixty-four pairs of Chinese SRCs and ORCs were constructed as the examples shown in (1a) and (1b). Each sentence contained two animate nouns or noun phrases, which were equally likely to be the patient or agent of the verb so that they were semantically reversible. Therefore, it was necessary to map thematic roles to the arguments for syntactic processing. Besides, a separate group of 20 students, who were naive to the purpose of the study and did not take part in any of the experiments, was asked to rate the naturalness of these sentences. The rating result showed that there was no significant difference in naturalness between the SRC and ORC conditions [*t*_1_(19) = 1.311, *p* = 0.205; *t*_2_(126) = 0.762, *p* = 0.448]. In addition to the target sentences, another 64 filler sentences with various structures (e.g., bàba zuótiān maile hìnduō haochī de shuiguo, ‘*yesterday the father bought a lot of delicious fruits*’) were also created as a control condition. All the pairs of SRCs and ORCs were evenly divided into two lists with an equal number of each type of sentences appearing in each list. Half of the participants received one list of the sentences, while the other half of the participants received the other list, so that one did not encounter both SRCs and ORCs in a pair. The order of the sentences in a sub-list was completely randomized across participants.

(1) The example of stimuli in Experiment 1
  (a) Subject-extracted relative clause (SRC)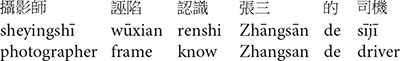    ‘The photographer framed the driver who knew Zhangsan.’
  (b) Object-extracted relative clause (ORC)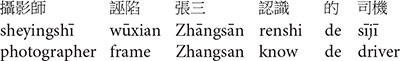    ‘The photographer framed the driver who Zhangsan knew.’

#### Procedure

**FIGURE 3:**
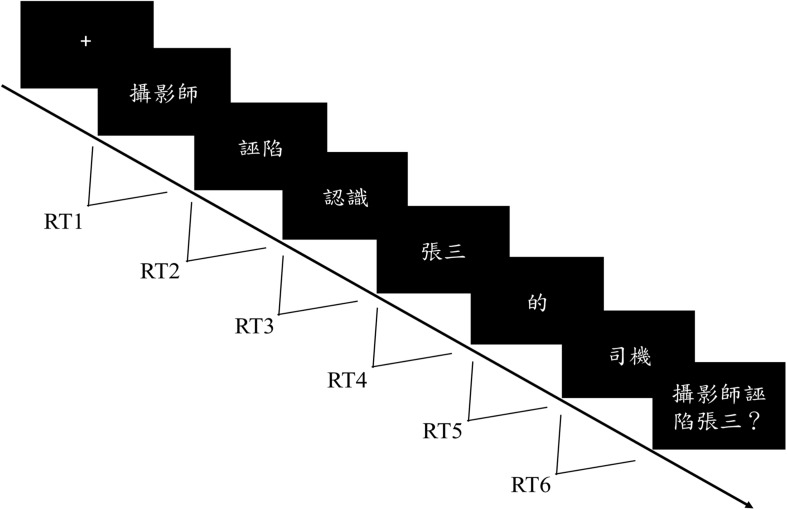
The experiment paradigm of Experiment 1. Each sentence was divided into six frames, each of which contained one to four characters, appearing in the center of the screen. Participants were instructed to carefully read the sentences, proceeding by pressing the space bar on a computer keyboard at their own pace. The amount of time the participants spent on each frame was recorded as the time between key-presses (RT1, RT2, RT3, RT4, RT5, RT6). After the presentation of the sentence, the participants were asked to make a true/false judgment in response to the probe question to determine whether they comprehend the meaning of the sentence.

Each participant was individually tested in a quiet and appropriately illuminated room. The whole experiment included four sessions (each contained eight target sentences for each type), and between sessions, participants could take a break. For each trial, a fixation cross first appeared on the center of the screen until the participants pressed the space bar to read the following sentence. Each sentence was divided into six frames, each of which contained one to four characters, appearing in the center of the screen. Participants were instructed to carefully read the sentences, proceeding by pressing the space bar on a computer keyboard at their own pace. The amount of time participants spent on each frame was recorded as the time between key-presses (RT1, RT2, RT3, RT4, RT5, RT6, as shown in Figure 3). After the final word of each sentence, a true/false comprehension question about the preceding sentence appeared on the computer screen. Feedback was displayed immediately when participants made their responses by pressing ‘F’ or ‘J’ to indicate true or false, respectively. Among all the sentences, the correct answers for half of the comprehension questions were true, while the correct answers for the other half were false. Afterward, a reminder to prompt participants to press the space bar to proceed to the next trial was shown in the center of the screen. Prior to the formal experiment, a practice with eight sentences was conducted to make sure that participants were familiar with the procedure. Stimuli presentation and data collection were programed via Python^1^.

#### Statistical Analysis

The online reading time of each frame of the sentences with correct responses to the offline comprehension questions was analyzed with the R software (R Development Core Team, 2011) using a mixed-effects model. The model was fit with the lme4 package (Bates et al., 2014) and the lmerTest package (Kuznetsova et al., 2014). The formula in R was

reading time∼sentence type+(1|subject)+(1|item)

The effect size of each significant difference, indicated by Cohen’s d, was further calculated based on the suggestions from Brysbaert and Stevens (2018). In addition to the word-by-word reading time, the total reading time of the two words in the RC structure was also obtained for further comparison. That is, the reading time of W3 + W4 was compared between the SRC and the ORC conditions (see similar practice in Gibson and Wu, 2013 and Vasishth et al., 2013). For the accuracy and reaction time of the offline comprehension questions, the conventional analysis of variance (ANOVA) was employed to determine the statistical relationship across sentence types.

### Results

#### Offline Comprehension Question Performance

**FIGURE 4:**
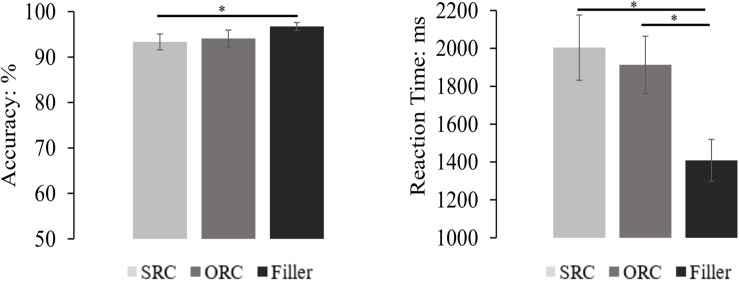
The comprehension performance of the RCs and the filler sentences. The results showed that the accuracy in the filler sentences was significantly higher than that in the SRC condition, but only numerically higher than that in the ORC condition. Moreover, there was also no significant accuracy difference between the SRC and the ORC conditions. On the other hand, the reaction time in answering the probe question of the filler sentence was significantly faster than that in the SRC and the ORC conditions, but no significant difference of the reaction time was found between SRCs and ORCs. The error bar indicates the 95% confidence interval. ^∗^*p* < 0.05.

The mean accuracy and reaction time (only from accurate responses and also after removal of the outliers beyond two standard deviations around the mean for each condition) of each sentence type were depicted in Figure 4. Generally, the comprehension accuracy of all trials was 94.72%, and that of target sentences was 93.72%. The accuracy difference between different types of sentences was significant in the by-subject analysis but not in the by-item analysis [*F*_1_(2,135) = 5.045, *MSE* = 29.050, *p* = 0.008; *F*_2_(2, 189) = 2.553, *MSE* = 79.882, *p* = 0.081]. Pairwise comparisons with Bonferroni correction showed that accuracy of filler sentences (96.74%) was significantly higher than that of the SRCs (93.34%, *p* = 0.009), and marginally higher than that of the ORCs (94.08%, *p* = 0.060), but there was no significant difference between the SRC and the ORC conditions (*p* = 1.000).

For the reaction time, the ANOVA found a significant difference among the three sentence types [*F*_1_(2,135) = 18.262, *MSE* = 0.259, *p* < 0.001; *F*_2_(2,121.237) = 38.172, *MSE* = 0.216, *p* < 0.001). Pairwise comparisons with Bonferroni correction showed that answering questions following filler sentences (1408 ms) was significantly faster than answering questions following the SRC sentences (2004 ms) and the ORC sentences (1912 ms) in both the by-subject and the by-item analysis (all *ps* < 0.001). Although the mean reaction time was numerically faster in the ORC sentences than the SRC sentences, the difference between them was far from significance (*ps* > 0.685).

#### Online Reading Time Performance

**FIGURE 5:**
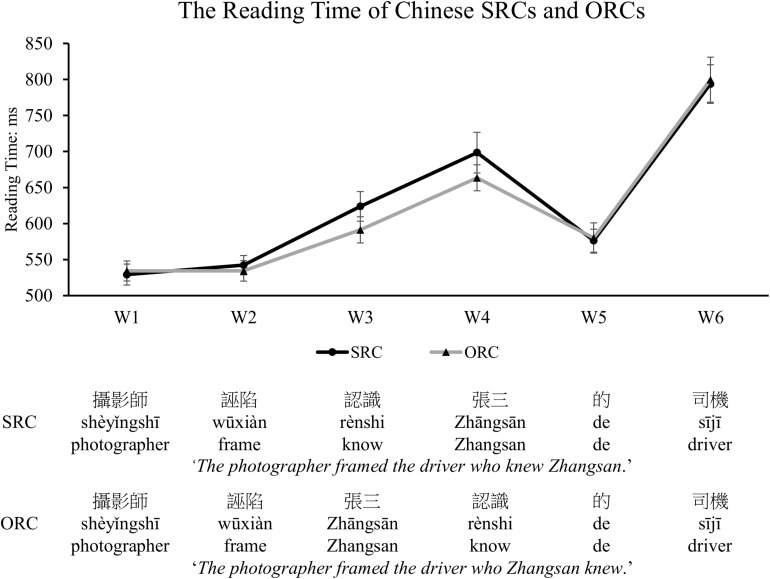
The reading time of each frame in the SRC and ORC conditions. Significantly longer reading time was found in the embedded clause region of the SRC condition compared with that of the ORC condition, which supports an ORC preference in Chinese. The error bar indicates the 95% confidence interval.

**FIGURE 6:**
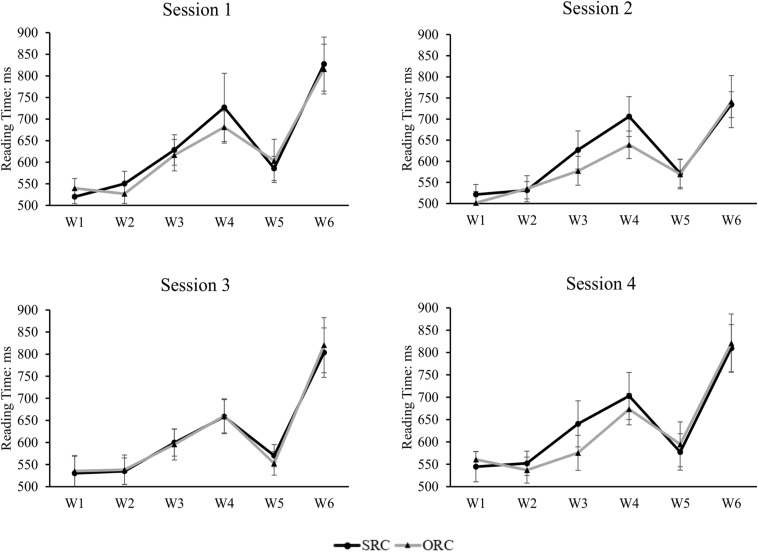
The reading time of each frame in the SRC and ORC conditions in each session. The results indicate that there is no significant preference difference across sessions, suggesting that the observed ORC preference does not develop due to exposure. The error bar indicates the 95% confidence interval.

Only those sentences whose comprehension questions were answered correctly were included for further analysis. The mean reading time of each frame and each type of target sentences after removal of outliers beyond two standard deviations around the mean in each condition (which included 5.3% of the raw data) was depicted in Figure 5 below. A mixed-effects model with sentence types as the fixed factor and subjects and items being modeled for random intercepts was applied to examine the significance of the effects of sentence types, different frames, and their interaction. The results showed that in addition to a significant main effect for frames (β = 0.04, *SE* = 0.002, *t* = 25.991, *p* < 0.001, Cohen’s *d* = 0.1014) and sentence types (β = −0.01, *SE* = 0.005, *t* = −2.737, *p* = 0.006, Cohen’s *d* = 0.0362), a significant interaction between sentence types and frames was also observed (β = 0.02, *SE* = 0.001, *t* = 19.11, *p* < 0.001, Cohen’s *d* = 0.0398). The significant reading preference for ORCs over SRCs was found in the embedded clause region (i.e., W3, β = −0.04, *SE* = 0.01, *t* = −3.065, *p* = 0.002, Cohen’s *d* = 0.0882; W4, β = −0.04, *SE* = 0.01, *t* = −2.693, *p* = 0.007, Cohen’s *d* = 0.0785). When combining the W3 and W4 as the whole one to represent the performance in the embedded clause region, we also observed a significant difference between SRCs and ORCs (i.e., W3 + W4, β = −0.04, *SE* = 0.01, *t* = −3.602, *p* = 0.0003, Cohen’s *d* = 0.0920). No statistical significance was observed in any other regions (W1, β = −0.0004, *SE* = 0.01, *t* = −0.045, *p* = 0.964; W2, β = −0.01, *SE* = 0.009, *t* = −1.183, *p* = 0.237; W5, β = 0.001, *SE* = 0.02, *t* = 0.088, *p* = 0.93; W6, β = −0.005, *SE* = 0.02, *t* = −0.271, *p* = 0.786). Further, the reading time differences between SRCs and ORCs in each session were depicted in Figure 6. The results indicated that no significant main effect was found in sessions (β = 0.02, *SE* = 0.02, *t* = 0.933, *p* = 0.351) and there was also no significant interaction between sentence types, frames and sessions (β = 0.002, *SE* = 0.003, *t* = 0.799, *p* = 0.424).

### Discussion

The results of Experiment 1 clearly showed a processing preference for ORCs over SRCs, which is in line with previous findings (e.g., Yang and Perfetti, 2006; Packard et al., 2010; Bulut et al., 2018). The observed ORC preference was also presented consistently across sessions, implying that the ORC preference might not develop due to exposure. Moreover, the results indicated a significantly faster reading time at the embedded clause region of the SRC condition compared to that of the ORC condition. One probable reason for this finding is the greater number of incomplete dependencies that need to be maintained in memory at the encounter of the embedded clause in the SRC than the ORC conditions, as proposed by the storage account of the DLT. Another potential reason might be due to the local ambiguity encountered at the embedded clause region, particularly in the SRC condition. Specifically, in the SRC condition, when participants encounter two verbs in successive W2 and W3 regions, there is temporarily ambiguous between an RC analysis and a main-clause analysis with a sequence of two verbs. On the other hand, in the ORC condition, only the interpretation of an RC analysis is activated. Thus, the additional demand is presumed to be required for this disambiguation in the SRC condition. It is not until the relativizer (i.e., *de*) in the SRC sentence is presented, which signals the reading of an RC structure, participants then realize that W3 is the embedded verb in an RC rather than the matrix verb. In other words, due to the cost for ambiguity resolution in the reading of SRCs, the significant difference of the reading time in SRCs and ORCs is more likely to be observed at the embedded clause region. To verify this speculation, we further conducted Experiment 2 to examine participants’ comprehension of SRCs and ORCs with an aspectual word ‘le’ added to the matrix verb.

## Experiment 2

One may concern that the observed ORC advantage in Experiment 1 might be (at least partially) attributed to increased ambiguity, hence processing load, when encountering two successive verbs in the SRC sentences. To minimize this concern, an aspectual marker ‘le’ was added to the matrix verb of Chinese object-modifying RC sentences. Moreover, the number of target sentences was reduced from 64 to 24 pairs to minimize the syntactic priming/learning effect if any and further examine whether the preference was still there. Besides, we excluded the usage of proper names as the noun phrases in the target sentences to avoid any difference that might be caused by specific properties of different kinds of noun phrases.

### Participants, Stimuli, and Procedure

A different group of 25 native Chinese-speaking students (10 females, aged from 21 to 27 years) from National Central University was recruited to determine the processing preference of Chinese RCs in this experiment. The same ethical standards as in Experiment 1 were applied. In this experiment, 24 pairs of RCs (24 SRCs, 24 ORCs) were created based on the materials of Experiment 1 (as shown in the example (2a) and (2b) below). The naturalness ratings from an independent group of 20 participants showed that there was no significant difference between the target sentences [*t*_1_(19) = −1.143, *p* = 0.267, *t*_2_(46) = 0.696, *p* = 0.490]. One might argue that some filler sentences with the canonical SVO structure, which shared the same word order with the ORC structure, might partially create the structural priming effect to drive the ORC advantage. To remove this potential concern, we selected 24 filler sentences with equal numbers of sentences starting with the Verb-Noun (VN) combination and sentences beginning with the Noun-Verb (NV) combination as a control condition. Among these 24 filler sentences, the first two words of eight sentences were the NV combination, which resembled the structure of ORCs, while the first two words of another eight filler sentences were the VN sequence, which resembled the structure of SRCs. The remaining eight filler sentences were with other various structures. The procedure and statistical analysis were the same as those described in Experiment 1.

(2) The examples of stimuli in Experiment 2
  (a) Subject-extracted relative clause with ‘le’ (SRC)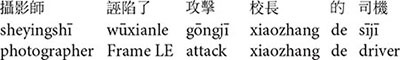    ‘The photographer framed the driver who attacked the principal.’
  (b) Object-extracted relative clause with ‘le’ (ORC)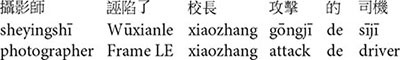    ‘The photographer framed the driver who the principal attacked.’

### Results

#### Offline Comprehension Question Performance

**FIGURE 7:**
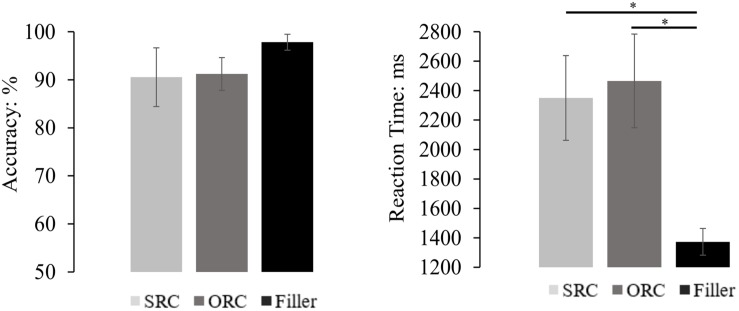
The comprehension performance of the SRC and the ORC conditions with an aspectual marker ‘le’ and also the filler sentences. The results showed that there was no significant accuracy difference across sentences, though the accuracy of the filler condition was numerically higher than that in the SRC and ORC conditions. On the other hand, the reaction time in answering the probe question of the filler sentence was significantly faster than that in the SRC and the ORC conditions, but no significant difference of the reaction time was found between SRCs and ORCs. The error bar indicates the 95% confidence interval. ^∗^*p* < 0.05.

The mean accuracy and reaction time (only from accurate responses and also after removal of the outliers beyond two standard deviations around the mean for each condition) of each sentence type were depicted in Figure 7. The results indicated that the accuracy difference among different types of sentence was significant [*F*_1_(2,72) = 3.134, *MSE* = 125.058, *p* < 0.050; *F*_2_(2,69) = 3.471, *MSE* = 112.234, *p* = 0.037]. However, pairwise comparisons with Bonferroni correction indicated that there was no significant accuracy difference between SRCs (90.54%) and ORCs (91.21%, *p* = 1.000). As for the reaction time, a significant difference was also found in both the by-subject and the by-item analysis [*F*_1_(2,44.329) = 18.413, *MSE* = 0.576, *p* < 0.001; *F*_2_(2,35.449) = 15.780, *MSE* = 0.417, *p* < 0.001]. Specifically, participants comprehended filler sentences significantly faster than comprehending the target sentences (all *ps* < 0.001), but the latency between SRCs (2466 ms) and ORCs (2350 ms) was still far from significance (*ps* = 1.000).

#### Online Reading Time Performance

**FIGURE 8:**
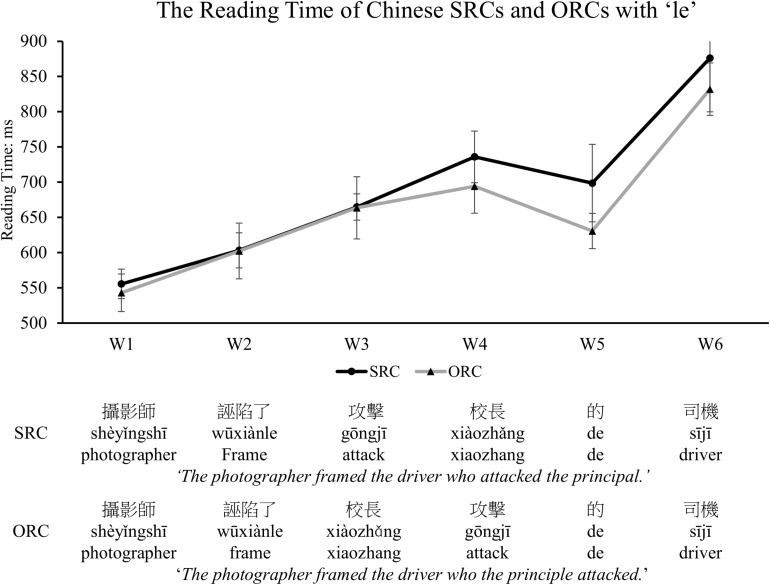
The reading time of each frame in SRCs and ORCs with an aspectual marker ‘*le.*’ With the assistance of the linguistic cue ‘*le*,’ the observed ORC preference was significantly reflected in the relativizer rather than the embedded clause region, supporting the prediction of the integration cost account. The error bar indicates the 95% confidence interval.

Only those sentences with correct responses to probed questions were included for further analysis. After removal of outliers beyond two standard deviations around the mean reading time in each condition (which excluded 5.6% of the raw data), we depicted the mean reading time of each frame for the SRC and the ORC sentences in Figure 8. The statistical results by using a mixed-effects model revealed that there was a significant main effect for frames (β = 0.05, *SE* = 0.003, *t* = 17.271, *p* < 0.001, Cohen’s *d* = 0.1565) and for sentence types (β = −0.04, *SE* = 0.01, *t* = −3.583, *p* = 0.0003, Cohen’s *d* = 0.1107). In addition, there was a significant interaction between sentence types and frames (β = 0.02, *SE* = 0.002, *t* = 10.97, *p* < 0.001, Cohen’s *d* = 0.05). As shown in Figure 8, Chinese SRCs were more difficult to comprehend than ORCs, significantly reflected in W5 (β = −0.08, *SE* = 0.03, *t* = −2.93, *p* = 0.004, Cohen’s *d* = 0.2291). No statistical difference was found in any other regions (W1, β = −0.01, *SE* = 0.01, *t* = −0.946, *p* = 0.345; W2, β = −0.007, *SE* = 0.02, *t* = −0.406, *p* = 0.685; W3, β = −0.009, *SE* = 0.02, *t* = −0.443, *p* = 0.658; W4, β = −0.04, *SE* = 0.02, *t* = −1.911, *p* = 0.06; W6, β = −0.05, *SE* = 0.034, *t* = −1.425, *p* = 0.155) or in the combination of the two words in the embedded clause region (W3 + W4, β = −0.03, *SE* = 0.02, *t* = −1.649, *p* = 0.10).

Considering that the materials employed in Experiment 1 and Experiment 2 were similar, thus we have conducted a cross-experiment analysis to examine whether the observed preference pattern was significantly reflected in different regions. Because the number of characters included in the W2 region in Experiment 1 (without ‘le’) and in Experiment 2 (with ‘le’) was not identical, the reading times were calculated based on a linear regression equation by taking the number of characters into consideration (Ferreira and Clifton, 1986). The results indicated that there was a significant difference between the two experiments (β = −0.02, *SE* = 0.08, *t* = −2.24, *p* = 0.025, Cohen’s *d* = 0.30), suggesting that the manipulation of the presence of ‘le’ did affect the comprehension of RC sentences.

### Discussion

The results from Experiment 2 also supported that Chinese ORCs were easier to comprehend than Chinese SRCs with tight control of the stimuli to remove any potential effects of noun phrases including proper names, syntactic priming/learning, and/or local ambiguity. Moreover, the presence of the aspectual marker ‘le’ seemed significantly influence the process of sentence comprehension, because it helped provide a syntactic cue to avoid the local ambiguity caused by the two successive verbs in the SRC sentences. The relativizer ‘de’ signaled the appearance of the RC structure, which demanded more integration cost for phrasal configuration, as reflected in the relativizer region, in the reading of SRCs than ORCs. Therefore, consistent with the prediction of the integration cost account of the DLT, the higher processing difficulty in SRCs than ORCs was significantly reflected in the relativizer ‘de’ in this experiment.

## General Discussion

The present findings from two self-paced reading experiments exhibit a consistent pattern that Chinese ORCs are easier to comprehend than Chinese SRCs. The ORC advantage in Chinese provides solid evidence in support of the DLT (Gibson, 1998), especially for the claim of the integration cost account that the processing difference between SRCs and ORCs arises at the point of dependency formation. The present behavioral results are also in line with the neuropsychological evidence that shows Chinese aphasic patients to exhibit selective difficulty in processing SRCs rather than ORCs (Su et al., 2007) and also consistent with the findings in the acquisition study of Chinese RCs (Chan et al., 2011).

As shown in Experiment 1 and Experiment 2, the same words are used at the beginning of the SRC and ORC conditions as the matrix subject and the matric verb, followed by a verb-noun-relativizer and a noun-verb-relativizer sequence, respectively. Thus, comparable processing costs, reflected in similar reading times, are predicted and observed before encountering RCs (see Figures 5, 8). During the reading of the following RCs, however, a greater integration cost is demanded for the processing of SRCs than ORCs, as reflected in increased reading time for the reading of SRCs. Such preference seems not to be completely driven by other potential confounds as raised in the previous literature (e.g., the effects of syntactic priming/learning and/or local ambiguity). Instead, the observed ORC preference is likely due to different memory cost involved. That is, increased processing difficulty, particularly at the relativizer and the head noun regions in reading a Chinese SRC with non-canonical word order, is thought to be due to the requirement of resources for the integration process across a longer filler-gap distance to understand the meaning of ‘who did what to whom’ than reading a Chinese ORC with canonical word order. Notably, the ORC advantage might even appear in the embedded clause before the relativizer region in object-modifying sentences, due to the local ambiguity encountered only in SRCs, as shown in Experiment 1. When the ambiguity is eliminated by a linguistic cue (i.e., the aspectual marker ‘le’ in Experiment 2), which informs readers of the upcoming elements for an RC reading, the processing advantage for Chinese ORCs over SRCs is significantly reflected in the relativizer region as predicted by the integration cost account of the DLT (see in Figure 8).

Taken together, the findings from the present study reveal that the self-paced reading time of Chinese ORCs is faster than that of Chinese SRCs, providing strong evidence in support of the DLT that emphasizes the contribution of working memory to sentence comprehension. Such a clear ORC preference in Chinese is consistent with the findings from previous behavioral and neurophysiological studies (Hsiao and Gibson, 2003; Yang and Perfetti, 2006; Packard et al., 2010; Yang et al., 2010; Gibson and Wu, 2013). On the other hand, the present results pose a challenge to the prediction of the structural distance account (O’Grady, 1997), which proposes that it is easier to relativize the subject than the object across languages. Moreover, the processing advantage for Chinese ORCs over SRCs is also in conflict with some previous studies (e.g., Lin and Bever, 2006) and the conclusion of Vasishth et al. (2013).

As reviewed and discussed earlier, several linguistic factors (including but not limited to structure ambiguity, animacy, thematic roles, previous experience) may affect comprehension of RCs. Also, as correctly pointed out by Vasishth et al. (2013), the lack of control of participants’ age and the testing environment could potentially contribute to the conflicting results reported in the literature. In the present study, in addition to controlling for these potential confounds as much as possible, the number of target sentences (64 sets) employed was relatively large. Because the processing difference between SRCs and ORCs is subtle and might be affected by multiple factors, lack of sufficient trials might be one of the reasons for the previously inconsistent findings. Specifically, most of the previous studies employed a relatively small number of target sentences: 24 sets in Hsiao and Gibson (2003) and Chen et al. (2008), 16 sets in Gibson and Wu (2013), 20 sets in Lin (2014). In these studies, the difference between SRCs and ORCs might be easily masked by some factors irrelevant to theoretical interests, such as attention, emotion, etc. Vasishth et al. (2013) tried to replicate the previous studies by adopting the same set of stimuli from Hsiao and Gibson (2003) and Gibson and Wu (2013), hence suffering the same problem of relatively few stimuli. The results from Vasishth et al. (2013) did not completely replicate previous findings, which again showed the unreliability of such experiments without sufficient stimuli. Recent endeavors to investigate Chinese RC processing from our lab all employed the relatively large number of target sentences as adopted in this study and have shown consistent support for the ORC preference in Chinese (Bulut et al., 2018; Xu et al., Unpublished). Therefore, we deem the adequate control of relevant factors and a sufficient number of target sentences helpful to increase the sensitivity to detect the subtle difference between SRCs and ORCs.

In the present study, the observed ORC preference is generally consistent across different sessions, suggesting that such processing asymmetry did not develop due to the short-time exposure to the target sentences. It should be noted, however, that our results should not be taken as direct evidence against the experience/frequency-based accounts or the statistical learning theory, which emphasize the importance of differential learning even with the same amount of exposure to SRCs and ORCs (MacDonald and Christiansen, 2002). Because we did not manipulate participants’ experience in reading RCs in this study, but only conducted a session-by-session comparison to observe the local learning effect within each participant, the design might not be optimal to reveal the effect of familiarity on sentence comprehension. We intentionally included many filler sentences to disguise the repetitive structure of SRCs and ORCs. Therefore, other research is still needed to examine the adequacy of the experience-based accounts.

In sum, our results suggest that the preference for SRCs or ORCs is language-specific and depends on the operations underlying sentence comprehension. In contrast to the theoretical approaches that predict a universal SRC advantage, our findings support that the preference of a specific RC structure may be diverse due to language-specific differences across languages. Such topological differences are not only observed in relative clause processing, but also have been reported in language-specific phoneme representation (Maddieson, 1984; Näätänen et al., 1997; Mielke, 2007), lexical access (Gerard and Scarborough, 1989), and also other syntactic rules (Evans and Levinson, 2009). For example, the four major word classes, that is, nouns, verbs, adjectives, and adverbs, have been often assumed to be essential in all languages. However, languages without an open adverb class (Hengeveld, 1992) or an adjective class (Enfield, 2004), or languages that lack a basic noun-verb distinction (Jelinek, 1995) have been found from the cross-linguistic data. Evans and Levinson (2009) also summarized cross-linguistic work to refute the assumed universality of the constituency, recursion, and grammatical relations. They thus further proposed that languages are much more diverse than unified in structure, and empirical data that demonstrate linguistic diversity should be the crucial evidence for theory development of the human mind. In the same vein, the present findings from Chinese highlight the necessity of cross-linguistic research and the importance of investigating language-specific processing mechanisms in sentence comprehension. Besides, further research is still needed to elucidate the complicated interplay among multiple factors (e.g., syntax, semantics, pragmatics) during the comprehension of Chinese RCs, which is out of the scope of the current study.

## Data Availability Statement

The raw data supporting the conclusions of this manuscript will be made available to any qualified researcher upon request.

## Ethics Statement

The studies involving human participants were reviewed and approved by Social and Behavioral Research Ethical Principles and Regulations of National Taiwan University. The participants provided their written informed consent to participate in this study.

## Author Contributions

KX, DH, and DW designed the experiment. KX and J-RD performed the data collection and analysis. KX and DW wrote the manuscript. KX, J-RD, DH, and DW were responsible for modifying the manuscript. All authors made their own contributions to the final manuscript and agreed to the submission of this version.

## Conflict of Interest

The authors declare that the research was conducted in the absence of any commercial or financial relationships that could be construed as a potential conflict of interest.

## Figures and Tables

**FIGURE A1:**
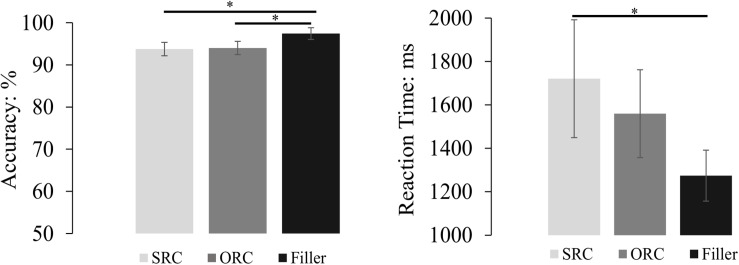
The comprehension performance of the subject-modifying RCs and filler sentences. The results showed that the accuracy in the filler sentence was significantly larger than that in SRCs and ORCs, but there was no significant accuracy difference between SRCs and ORCs. On the other hand, the reaction time in answering the probe question of the filler sentence was significantly faster than that in SRCs but only numerically faster than that in ORCs. Moreover, no significant difference of the reaction time was found between SRCs and ORCs. The error bar indicates the 95% confidence interval. ^∗^*p* < 0.05.

**FIGURE A2:**
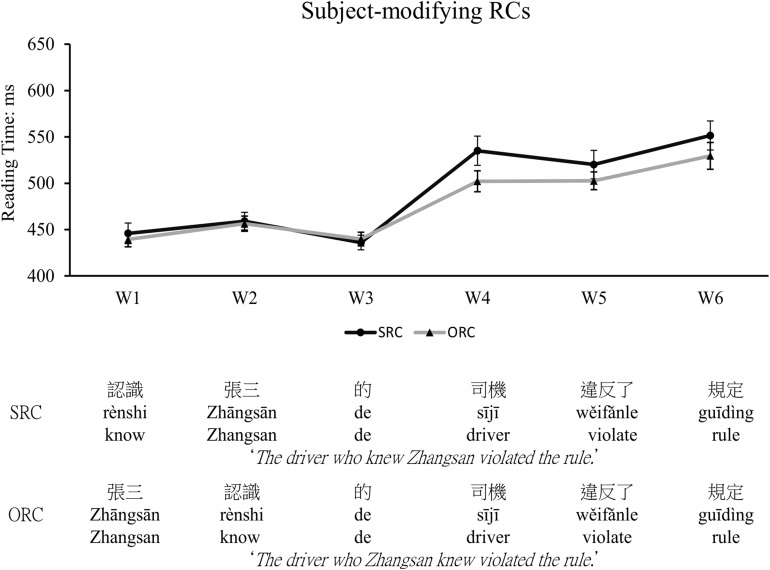
The reading time of each frame in the SRC and ORC sentences. Significantly longer reading time was found in the head noun region of the SRC sentences compared with that of the ORC sentences, which indicated a clear ORC preference in Chinese.

## Footnotes

1 https://www.python.org

## Appendix: The Experiment with Chinese Subject Modifying SRCs and ORCs

### Methods

Forty-six native Chinese-speaking students (22 females, aged from 20 to 29 years) from National Central University participated in this experiment. The sample materials are as shown in below. The procedure and the statistical analysis was the same as described in main texts. Moreover, for subject-modifying sentences, the reading time of W1 + W2 was compared between SRC and ORC sentences (see similar practice in Gibson and Wu, 2013; Vasishth et al., 2013).

(a) Subject relative clause at subject-modifying position (SRC)
‘The driver who knew Zhangsan violated the rules.’
(b) Object relative clause at subject-modifying position (ORC)
‘The driver who Zhangsan knew violated the rules.’

### Results

#### Offline Comprehension Question Performance

The mean accuracy and reaction time (only from accurate responses and also after removal of the outliers beyond two standard deviations around the mean for each condition) of each sentence type were illustrated in Appendix Figure A1. Generally, the comprehension accuracy of all trials and the target sentences was 95.06 and 93.88%, indicating that those sentences were natural to comprehend as confirmed by the rating results. Because the accuracy of sentences violated the Levene’s test of homogeneity of variances, a Welch analysis of variance was employed to determine the statistical relationship among the accuracy of different sentence types. The result showed that there was a significant difference between different types of sentences [*F*_1_(2,135) = 6.879, *MSE* = 27.941, *p* < 0.001; *F*_2_(2,113.276) = 7.619, *MSE* = 51.829, *p* = 0.001]. *Post hoc* comparison with Bonferroni correction further indicated the accuracy of filler sentences (97.42%) was significantly higher than that of target RC sentences (all *ps* < 0.007), but no significant difference between the SRC sentences (93.75%) and the ORC sentences (94.02%) was found in either the by-subject or by-item analysis (*ps* = 1).

For the reaction time, the ANOVA found a significant difference among the three sentence types [*F*_1_(2,135) = 4.519, *MSE* = 0.266, *p* = 0.013; *F*_2_(2,122.008) = 36.428, *MSE* = 0.068, *p* < 0.001]. Pairwise comparisons with Bonferroni correction showed that answering questions following filler sentences (1274 ms) was significantly faster than answering questions following the SRC sentences (1720 ms) in both the by-subject and the by-item analysis (*ps* < 0.013), but was only significantly faster than answering questions following the ORC sentences (1572 ms) in the by-item analysis (*p* < 0.0001) but not in the by-subject analysis (*p* = 0.103). Although the mean reaction time was numerically faster in the ORC sentences than the SRC sentences, the difference between them was far from significance (*ps* > 0.272).

#### Online Reading Time Performance

Only those sentences whose comprehension questions were answered correctly were included for further analysis. The mean reading times of frame 1 to 6 for the SRC and the ORC conditions were depicted in Appendix Figure A2 after removal of outliers beyond two standard deviations around the mean in each condition, which excluded 4.9% of the raw data. A mixed-effects model with sentence types as the fixed factor and subjects and items being modeled for random intercepts and slopes was applied to examine the significance of the effects of sentence types, different frames, and their interaction. The statistical results indicated that there was a significant interaction between sentence types (SRC and ORC) and frames (β = 0.01, *SE* = 0.001, *t* = 19.49, *p* < 0.001), as well as significant main effects for frames (β = 0.02, *SE* = 0.001, *t* = 21.996, *p* < 0.001) and sentence types (β = −0.01, *SE* = 0.004, *t* = −2.752, *p* = 0.008). Furthermore, as shown in Figure 5, the ORC sentence was found to read significantly faster than the SRC sentence at the head noun region (W4, β = −0.03, *SE* = 0.01, *t* = −2.68, *p* = 0.007), but no significant difference between the SRC and the ORC sentences was found within the embedded clause region (W1, β = −0.01, *SE* = 0.01, *t* = −1.063, *p* = 0.291; W2, β = −0.002, *SE* = 0.006, *t* = −0.283, *p* = 0.78; W1 + W2, β = −0.004, *SE* = 0.005, *t* = −0.882, *p* = 0.378) or at the relativizer region (W3, β = 0.005, *SE* = 0.005, *t* = 0.933, *p* = 0.351) or the post-head positions (W5, β = −0.02, *SE* = 0.01, *t* = −1.737, *p* = 0.0856; W6, β = −0.02, *SE* = 0.01, *t* = −1.853, *p* = 0.064).

### Discussion

The results of this subject-modifying experiment also indicated that participants had better performance in understanding Chinese ORCs than SRCs. Such an ORC preference was significantly reflected at the head noun region during sentence reading, which replicated some previous findings (Chen et al., 2008; Qiao et al., 2012; Gibson and Wu, 2013) and was most consistent with the prediction of the integration cost of the DLT. On the other hand, the storage cost account was not supported by the findings of this experiment, as no significant difference was observed within the embedded clause region. However, as claimed in Gibson and Wu (2013), because of temporary ambiguity, SRCs may be read more slowly than ORCs initially, because there are more predicted syntactic heads in the initial analysis of the first part of SRCs than in the initial part of ORCs. Thus, the observed preference could be predicted either by the integration cost or by ambiguity resolution, which makes it less interpretable than the results of the two experiments in the main text.
